# GNA15 predicts poor outcomes as a novel biomarker related to M2 macrophage infiltration in ovarian cancer

**DOI:** 10.3389/fimmu.2025.1512086

**Published:** 2025-02-07

**Authors:** Qin Liu, Yabing Sun, Tao Zhang, Wanrun Lin, Jing Zhang, Huijuan Zhang, Wenxin Zheng, Hong Xu, Feng Zhou

**Affiliations:** ^1^ Department of Pathology, The International Peace Maternal and Child Health Hospital, School of Medicine, Shanghai Jiao Tong University, Shanghai, China; ^2^ Department of Pathology, Zhejiang University School of Medicine Women’s Hospital, Hangzhou, Zhejiang, China; ^3^ Department of Gynecology, The International Peace Maternal and Child Health Hospital, School of Medicine, Shanghai Jiao Tong University, Shanghai, China; ^4^ Shanghai Key Laboratory of Embryo Original Diseases, Shanghai, China; ^5^ Department of Gynecology, Zhejiang University School of Medicine Women’s Hospital, Hangzhou, Zhejiang, China; ^6^ Laboratory of Pathology, National Cancer Institute, National Institutes of Health, Bethesda, MD, United States; ^7^ Department of Biological Sciences, College of Arts and Sciences, University at Albany, State University of New York (SUNY), Albany, NY, United States; ^8^ Department of Pathology, Department of Obstetrics and Gynecology, University of Texas Southwestern Medical Center, Dallas, TX, United States

**Keywords:** ovarian carcinoma, cancer microenvironment, tumor-associated macrophages, prognostic model, GNA15

## Abstract

**Background:**

The exploration of genetic signatures within the ovarian cancer (OC) tumor microenvironment (TME) remains limited. M2-like tumor-associated macrophages (M2-like TAMs) are pivotal in OC progression and therapy. This study aims to establish a novel prognostic signature and identify M2-like TAM-related biomarkers in OC using RNAseq-based transcriptome analysis.

**Methods:**

Prognostic M2-like TAM-related genes were identified through univariate Cox regression, consensus clustering, and LASSO regression. Immune landscape analysis was conducted to assess immune cell composition and immune checkpoint genes in high- and low-risk groups. Subsequently, *in vitro* cell experiments and OC cohorts were performed.

**Results:**

Gene set enrichment analysis revealed that GNA15 is involved in immune responses like leukocyte transendothelial migration and FcγR-mediated phagocytosis. GNA15 was up-regulated in cisplatin-resistant OC cells, and its *in vitro* down-regulation decreased cell proliferation. An eight-gene prognostic model, including M2-like TAM-related genes, independently predicted poor outcomes in OC. GNA15 emerged as a hub gene positively correlated with M2-like TAMs infiltration, predicting unfavorable outcomes across OC cohorts. Moreover, GNA15 expression correlated positively with CD163 expression, suggesting its role in macrophage polarization.

**Conclusion:**

GNA15 plays an immunosuppressive role in OC progression linked to M2-like TAMs polarization and stands as a potential prognostic marker in OC.

## Background

Ovarian cancer (OC) is the leading cause of reproductive cancer-related deaths in women globally (1). Its aggressive nature results in a low early detection rate, with 60%-70% of patients diagnosed at advanced stages (2). Treatment typically involves surgery combined with chemotherapy, and targeted therapies, yet resistance to chemotherapy remains a significant challenge leading to treatment failures. Given the modest gains in survival rates with conventional therapies, there is increasing interest in exploring immunotherapy as a viable treatment strategy. Thus, understanding the tumor-immune interactions and identifying novel therapeutic targets are critical for improving ovarian cancer outcomes.

The tumor microenvironment (TME) comprises a complex network involving tumor cells, immune cells, stromal elements, and various signaling molecules such as cytokines and chemokines. Extensive research has highlighted the TME’s role in tumorigenesis, cancer progression, and treatment resistance (3–5). Tumors can modulate the microenvironment to promote growth, metastasis, or evade therapies (6, 7). Moreover, the TME plays a pivotal role in regulating immune responses in cancer, influencing both tumor suppression and progression (8).

Among the key players in the TME are tumor-associated macrophages (TAMs), which are highly plastic immune cells within the tumor microenvironment, capable of adopting distinct M1 or M2 phenotypes in response to local signals (9, 10). M1-type macrophages exert anti-tumor effects, while M2-type macrophages can promote tumor progression by enhancing proliferation and invasion (11, 12). The interactions between TAMs, tumor cells, stromal cells, and endothelial cells are pivotal in reshaping the TME, thereby facilitating tumor growth, immune evasion, and metastasis (13, 14). Due to the dual polarization of TAMs, with distinct pro-tumor (M2) and anti-tumor (M1) phenotypes, modulating their polarization presents a promising therapeutic strategy for cancer treatment.

Guanine nucleotide-binding protein subunit alpha-15 (GNA15) was characterized as one of the hub genes most associated with M2-like TAMs infiltration in OC. It has been reported that GNA15 mediates a non-classical G protein-coupled receptor (GPCR) signaling pathway that, through CD312, promotes a suppressive TME in the onset and progression of pediatric acute lymphoblastic leukemia (15). In macrophages, genetic deletion of GNA15 almost completely blocked C5a-induced Ca2+ transients, but chemotaxis and cell spreading were preserved (16). These findings offer insights into the expression and function of the GNA15 gene in macrophages, indicating that it may play a crucial role in macrophage signal transduction and cellular behavior. Previous studies have also highlighted the involvement of GNA15 in various tumor types such as liver cancer, pancreatic adenocarcinoma, acute myeloid leukemia, and ovarian cancer (17–21). Moreover, GNA15 has been implicated in the TME in pancreatic cancer and melanoma (22, 23). Despite its involvement in other cancers, GNA15’s role in the OC microenvironment remains poorly understood.

Our study aims to investigate the association between GNA15 and M2-like TAMs in OC. We developed a prognostic model based on M2-like TAMs to predict OC patient survival rates and have conducted comparative analyses of immune landscapes across different risk groups.

## Materials and methods

### Dataset acquisition and preprocessing

This study included patients from five OC cohorts namely GSE65986, GSE3149, GSE63885, GSE140082 as well as TCGA-OC. Gene expression data, corresponding clinical and survival information of ovarian cancer were download from the Genomic Data Commons (GDC, https://portal.gdc.cancer.gov/) and Gene Expression Omnibus (GEO) dataset (https://www.ncbi.nlm.nih.gov/geo/). The mRNA-seq data were converted to TPM (transcripts per million) values and normalized by log2(x+1) for following analyses.

### Obtainment of prognostic M2-like TAM-related genes

To identify genes strongly associated with M2 macrophage infiltration, we employed the R WGCNA package to construct mRNA co-expression networks based on TCGA-OC gene expression data (24). Initially, a similarity matrix was generated by calculating the Pearson correlation coefficient between each pair of genes, following transformed to an adjacency matrix by WGCNA. By selecting a soft thresholding power (β=5) and setting a network merge height of 0.25 to combine similar gene modules, a total of 31 gene modules were obtained. Subsequently, the gene module including 113 hub genes that showed the highest correlation with M2-like TAM infiltration was extracted for further analysis by performing Pearson’s correlation analysis. Finally, we verified 18 M2-like TAM-related genes with prognostic value using univariate Cox regression analysis on the 113 hub genes.

### Consensus clustering analysis based on M2-like TAM infiltration

We performed consensus clustering based on the 18 prognostic M2-like TAM-related genes using R Consensus Cluster Plus package (25) for TCGA-OC datasets. According to the cluster consensus value, the optimal K value was set as 2 and the pheatmap tool in R was used to create the cluster map.

### Functional enrichment analysis, immune cell infiltration analysis and genomic mutations analysis between two clusters

We screened the differentially expressed genes (DEGs) between C1 and C2 subgroups using the limma package in R software. Kyoto Encyclopedia of Genes and Genomes (KEGG) analyses were conducted to explore the biological pathway of the DEGs. Then, Gene Set Enrichment Analysis (GSEA, https://www.gsea-msigdb.org/) was performed to analyze functional pathways enriched between C1 and C2 subgroups. Pathways with a P value <0.05 and a false discovery rate <0.25 were considered significantly enriched pathways. To identify differences in the immune landscape between C1 and C2 subgroups, we loaded the expression data of TCGA-OC into CIBERSORT (https://cibersort.stanford.edu/). To compare the genomic mutations between two clusters, OC somatic mutation data were obtained from the TCGA Genomic Data Commons Data Portal. The *Maftools* package in R software was used to visualize the mutations between C1 and C2 subgroups.

### Development and verification of the M2-like TAM-related prognostic model

Totally, 18 prognostic M2-like TAM-related genes were identified. Least absolute shrinkage and selection operator (LASSO) regression analyses and tenfold cross-validation was used to determine the penalty regularization parameter lambda to develop an eight M2-like TAM -related genes (ALOX5AP, CCR1, GNA15, IL2RG, ITGAM, LPXN, MSR1, and PDCD1LG2) prognostic model. Based on the best lambda values and the corresponding coefficients, the riskscore of each patient was calculated by using the score formula as follows: Riskscore=(0.1245)*ALOX5AP+(-0.1815)*CCR1+(0.1256)*GNA15+(-0.1901)*IL2RG+(0.0947)*ITGAM+(-0.0654)*LPXN+(0.1384)*MSR1+(-0.1126)*PDCD1LG2. Then, patients were divided into low- and high-risk groups based on the optimal cutoff of the riskscore. To compare the overall survival (OS) time between high and low-risk groups, we conducted Kaplan-Meier analysis and the log-rank test. To further validate the prognostic model, GSE140082 was select as the validation cohort to evaluate the M2-like TAM-related prognostic model constructed based on TCGA database.

### Expression of GNA15 in normal and tumor samples

Gene Expression Profiling Interactive Analysis 2 (GEPIA2) is an online tool for analyzing RNA-sequencing expression data between normal and tumor samples (26). Here, we compared GNA15 expression in 33 types of tumor by GEPIA2.

### TIMER 2.0 analysis

The association between GNA15 expression and the M2-like TAM infiltration or M2 macrophages gene markers expression was explored by Tumor Immune Estimation Resource 2.0 (TIMER 2.0; http://timer.comp-genomics.org/), which is a valuable tool that can systematically evaluates the infiltration of various immune cells (27).

### *In vitro* cell experiments

IOSE80, SKOV3, TOV112D, MDAH2774, OVCAR5 and OVCAR8 cell lines (ATCC, USA) were used for *in vitro* experiments. In addition, A2780-cis and SKOV3-cis were cisplatin-resistant cell lines (ATCC, USA) for validation of GNA15 involved in drug resistance. All cells were cultured in RPMI 1640 medium containing 10% FBS at 37°C with 5% CO2. ShRNA-mediated knockout is a transcriptional-level gene suppression, while the gRNA-guided CRISPR/Cas9 system is typically used for permanent gene knockout by introducing mutations or deletions. To ensure the reliability of the experimental data following gene knockout, we used both shRNA and gRNA-guided CRISPR/Cas9 methods to knockout the GNA15 gene. GNA15 was knocked down using both shRNA and CRISPR/Cas9. The sequences for Sh2, Sh3, and gRNA were as follows: CCTCGCATTGTTTGGGACTAT, CCATTGTTTCGAGAACGTGAT, and CGATCACGTTCTCGAAACAA, respectively. The Cell Counting Kit-8 (Boster, China) was used to assess cell proliferation according to the manufacturer’s instructions.

### Clinical sample collection

In this study, 60 OC and 30 normal ovarian paraffin embedded samples, 12 OC and 9 normal control samples (fresh tissue) from 2003 to 2017 were collected. The sample collection procedure and further studies were approved by the institutional review board. Patient clinicopathologic information, including age, clinical stage, and follow-up data were extracted from the electronic clinical information system database. Hematoxylin and eosin and immunohistochemistry (IHC) slides were reviewed by the gynecological pathologist (F.Z.) and the pathologic diagnosis was confirmed. The pathological diagnosis for all patients is high-grade serous carcinoma. Subsequently, IHC and Quantitative real-time PCR (qRT-PCR) were performed on these samples. All experiments were conducted following the guidelines and instructions approved by the manufacturer.

### IHC

Paraffin sections (4-µm) were stained with the antibodies using the 2-step Envision method according to the manufacturer’s instructions and visualized using 3-diaminobenzidine tetrachloride (Sigma, St Louis, MO). Antibodies against GNA15 (1:350 dilution; NBP2-16557, Novus), CD163 (1:100 dilution, Gene, China) were used in this study. The detection kit was obtained from Dako Corporation (Glostrup, Denmark). The negative control entailed the use of the same non-specific IgG but omitting the primary antibody. Positive cells were indicated by the presence of yellow to brown DAB staining in the nucleus or cytoplasm. GNA15 and CD163 expression were quantified in IHC by evaluating both the staining intensity and the percentage of positively stained cells. Staining intensity was scored as none, weak, moderate, or strong, and the percentage of positive cells was estimated. The score was then calculated by multiplying the staining intensity with the percentage of positive cells, providing a semi-quantitative measure of protein expression.

### qRT-PCR

The expression level of GNA15 was evaluated by qRT-PCR. TRIzol reagent (15596018, Invitrogen) was used to extract total RNA. The integrity and fragment size of the extracted RNA were assessed through 1% agarose gel electrophoresis. Additionally, the quality of the extracted RNA was measured using NanoDrop 2000 (Thermo Scientific, USA). qRT-PCR was performed to detect GNA15 mRNA expression in cancer cell lines and fresh cancer tissues. Primers sequences of GNA15 and GAPDH were as follows: GAPDH, TTCACCACCATGGAGAAGGC and GGCATGGACTGTGGTCATGA; and GNA15, CCCTGGTTCAAAAGCACATCCG and AACCTCTTGGCTGCCTCAGCAT, respectively.

### Statistical analysis

Kaplan-Meier analysis and log-rank tests were conducted to compare survival differences between different subgroups. Multivariate and univariate cox regression analyses were applied to identify prognostic factors. Correlation analysis between GNA15 and other factors were conducted using Pearson or Spearman correlation analyses. All statistical analyses were conducted using R software. *P*<0.05 was considered statistically significant.

## Results

### Identification of prognostic M2-like TAM-related genes by WGCNA in OC

**Figure 1:**
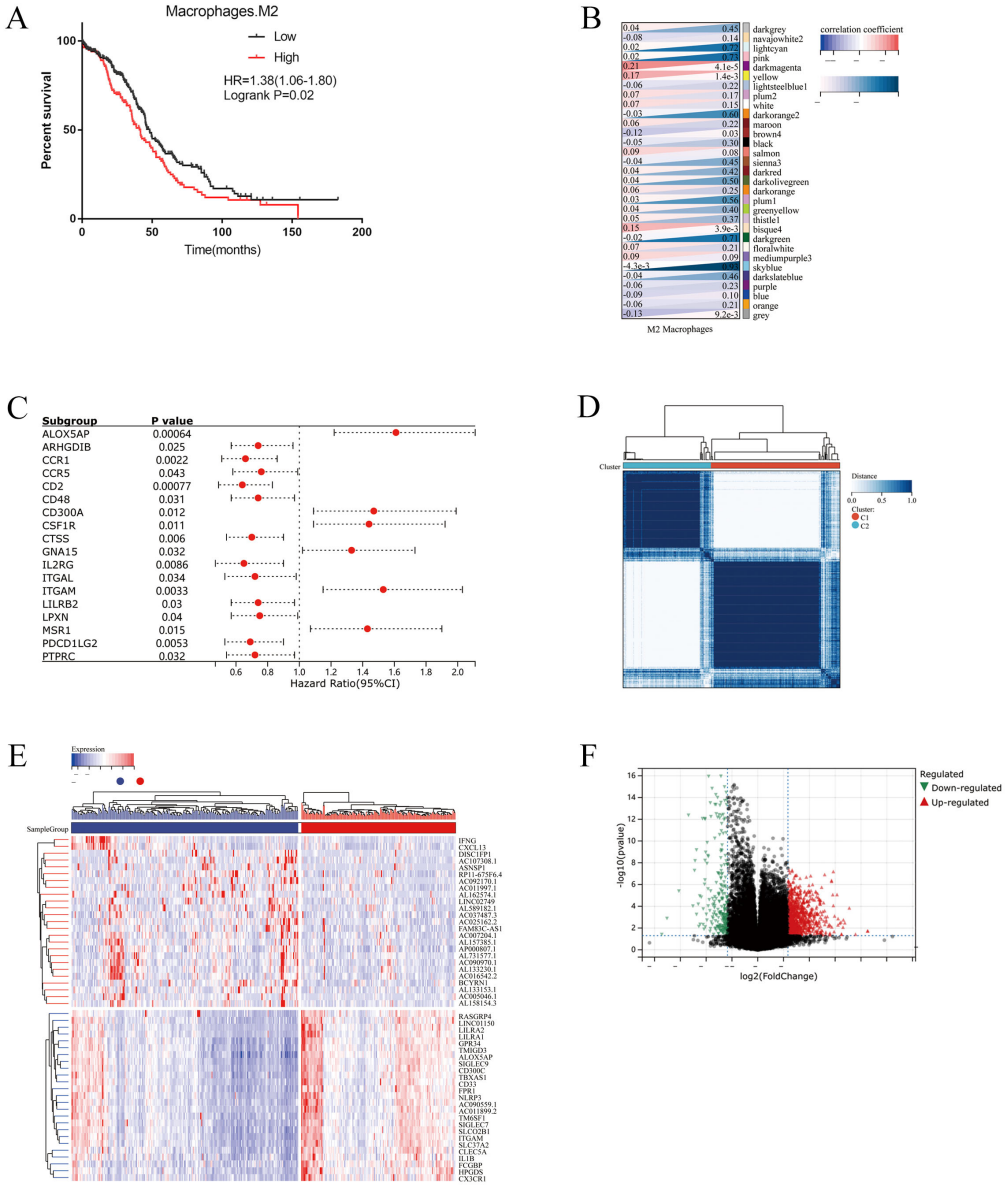
Identification of prognostic M2-like TAMs related genes. **(A)** Kaplan–Meier analysis showing the correlations between M2-like TAMs infiltration and overall survival (OS) in TCGA OC cohorts. Patients were grouped into “high” or “low” groups based on the median CIBERSORT-based M2 macrophages score. **(B)** Weighted correlation network analysis (WGCNA) identifies M2-like TAMs infiltration correlated modules. **(C)** Univariate COX regression analysis for the 18 genes associated with the infiltration of M2 macrophages. **(D)** Consensus clustering showed that 2 clusters were most stable. **(E)** Heatmap plot exhibiting the up-regulated and down-regulated genes in OC tissues between Cluster1 and Cluster2. **(F)** Volcano plot showing differential expressed genes in two clusters.

Totally, 22 kinds of immune cell infiltration were calculated by the CIBERSORT algorithm based on TCGA-OC datasets. Kaplan–Meier analyses showed that high M2 macrophage infiltration indicated a poor prognosis (**Figure 1A**). Based on the results, we performed WGCNA to detect the modules related to M2 macrophage infiltration. A soft threshold power of β = 5 (scale-free R2 = 0.90) was selected to build a scale-free network (**Supplementary Figures S1A–E**). As a result, 31 modules were generated by WGCNA. Among all the modules, the darkmagenta module was the most significantly related to M2 macrophages according to correlation analysis (correlation = 0.21, *P*<0.001, **Figure 1B**). Then, univariate cox regression analyses identified 18 prognostic M2-like TAM-related genes from the darkmagenta module (**Figure 1C**).

### Consensus clustering based on prognostic M2-like TAM-related genes and following pathway analysis

**Figure 2:**
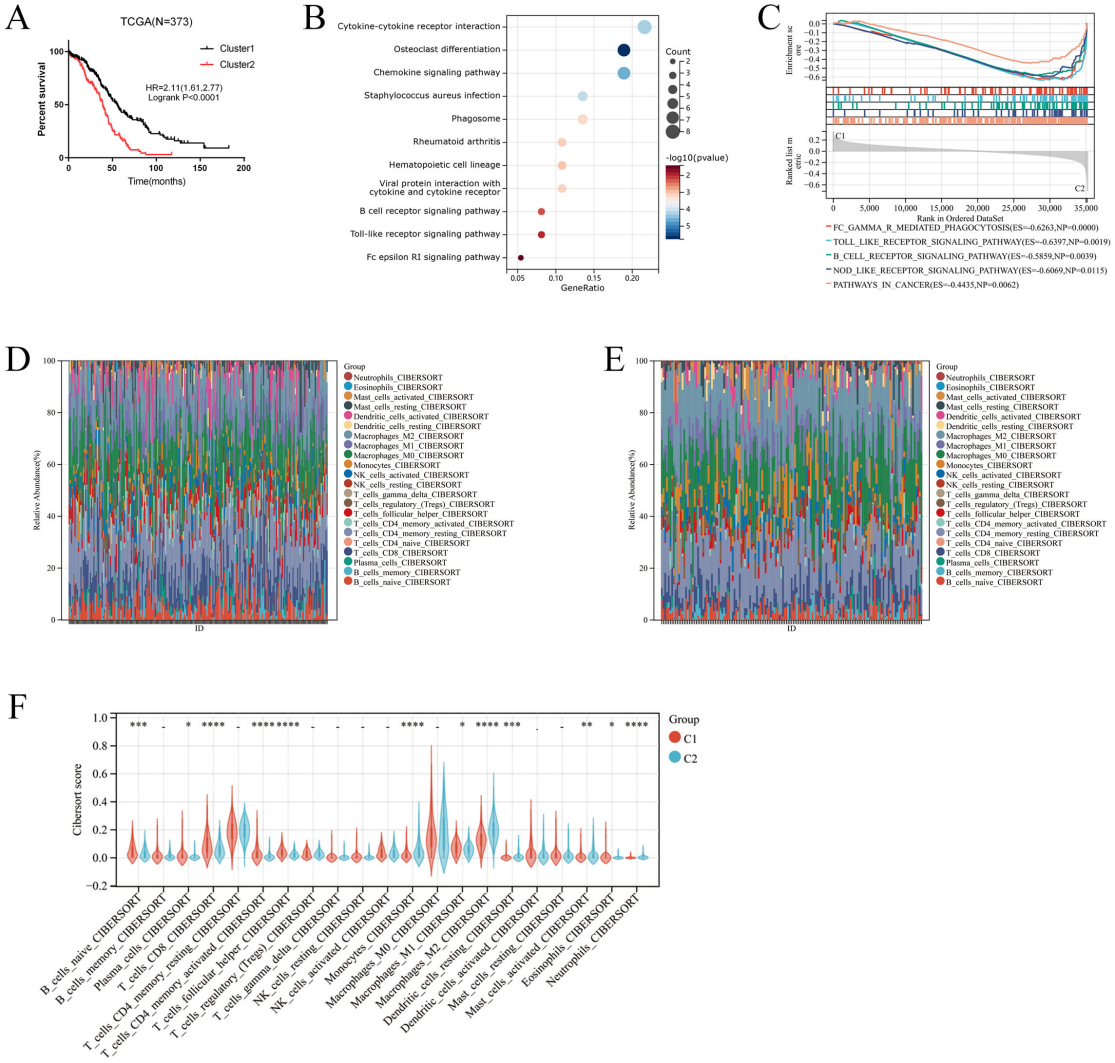
Identification of M2-like TAMs related cluster. **(A)** Kaplan–Meier analysis showing overall survival (OS) of two clusters. **(B)** KEGG analysis for the differential expressed genes in two clusters. **(C)** GSEA analysis for the two clusters. **(D-F)** The comparison of the immune cells infiltration between the two clusters. *p < 0.05; **: p < 0.01; *** p < 0.001; ****p < 0.0001.

**Figure 3:**
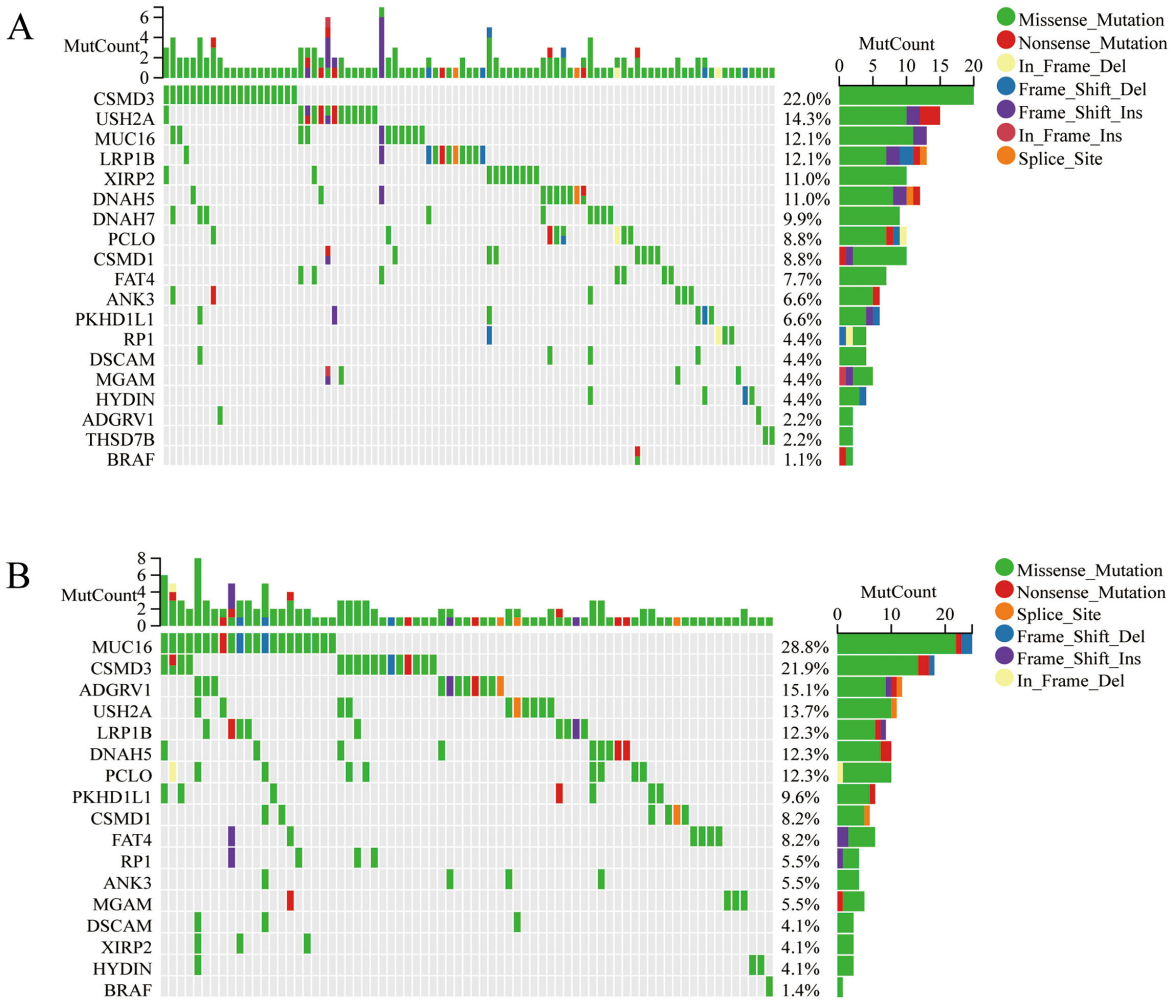
The mutation analysis of two clusters. The waterfall plot shows the top 20 genes with mutation frequency of Cluster1 **(A)** and Cluster2 **(B)**.

Two clusters were identified by R Consensus Cluster Plus package for Consensus Clustering in TCGA-OC dataset based on the 18 prognostic M2-like TAM-related genes: Cluster1 (222 cases) and Cluster2 (151 cases) (**Figure 1D**). A total of 123 DEGs were visualized with the R package “heatmap” and the volcano maps (**Figures 1E, F**). Compared to Cluster1, Cluster2 showed a significantly worse prognosis (log-rank *P*<0.0001, **Figure 2A**). KEGG showed that DEGs between Cluster1 and Cluster2 were mainly enriched in Cytokine-cytokine receptor interaction, Osteoclast differentiation, Phagosome, B cell receptor signaling pathway, Toll-like receptor signaling pathway and Fc epsilon RI signaling pathway (**Figure 2B**). GSEA results showed that immune related pathways were mainly enriched in Cluster2 (**Figure 2C**). Accordingly, the infiltration of M2 macrophage is significantly higher while infiltration of B cells naive, plasma cells, T cells CD8, T cells CD4 memory activated and T cells follicular helper is significantly lower in Cluster2 than that in Cluster1 (**Figures 2D–F**). In addition, somatic mutations analysis showed that mutations frequency in Cluster2 is significantly higher than that in Cluster1 (**Figures 3A, B**).

### Construction of prognostic model based on prognostic M2-like TAM-related genes

**Figure 4:**
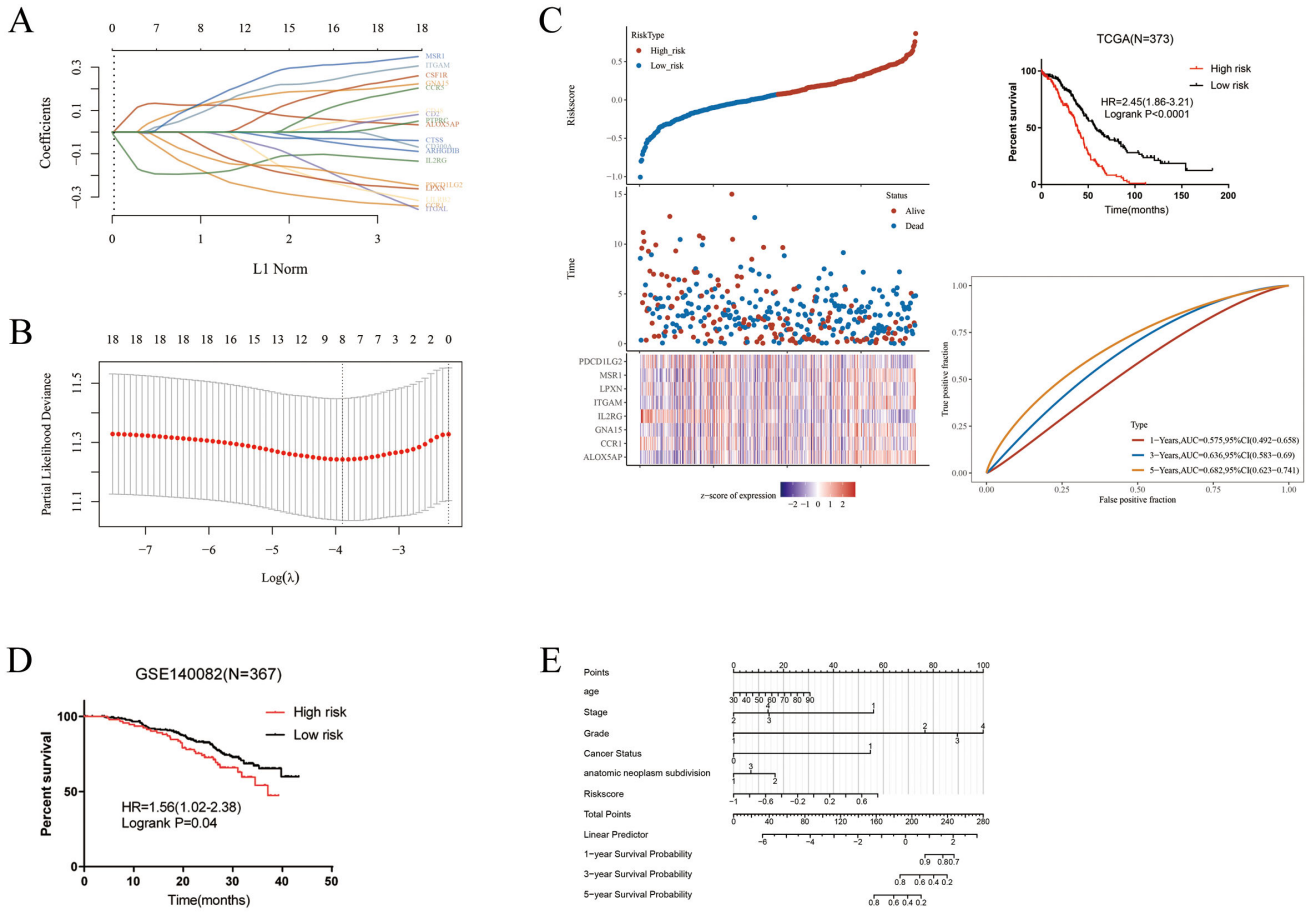
Construction of a risk model. **(A–C)** LASSO analysis for M2-like TAMs related genes associated with the survival rate of OS. **(D)** Validation of the prognostic model in GSE140082. **(E)** Age, Stage, riskscore, grade, cancer status and anatomic neoplasm subdivision were used to construct the nomogram.

A LASSO analysis was performed to develop a prognostic model, and an 8-M2-like TAM-related gene signature was established (**Figures 4A, B**). Based on the optimal cut-off of riskscores, the patients were classified into the high and low-risk groups. Kaplan-Meier analysis showed that patients in the high-risk group had a worse prognosis than that in the low-risk group (**Figure 4C**). Furthermore, GSE140082 was used as the independent cohort and verified the reliability of the prognostic model (**Figure 4D**). Using the riskscore from our prognostic signature along with other clinicopathological factors, we developed a nomogram to provide a more comprehensive prediction of patient survival (**Figure 4E**).

### High-and low-risk groups exhibit different immune cell infiltration and immune gene expression

**Figure 5:**
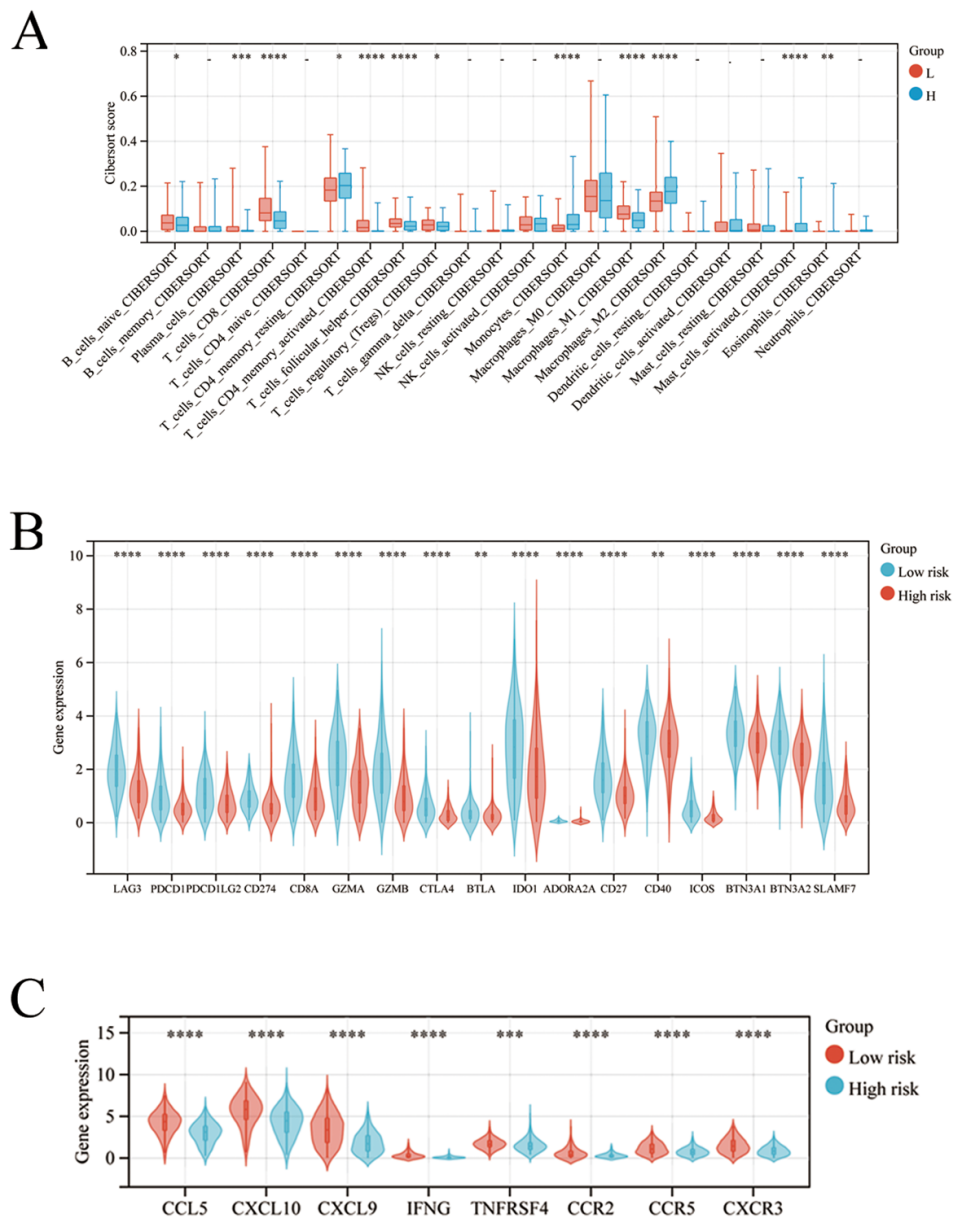
High-and low-risk groups exhibit different immune cell infiltration and immune gene expression. **(A–C)** The comparison of the immune cells infiltration and expression of immune checkpoint-related genes between the high-and low-risk group. *p < 0.05; **: p < 0.01; *** p < 0.001; ****p < 0.0001.

To explore the underlying immune-related factors difference between high- and low-risk groups, we compared immune cell infiltration and immune gene expression in the two groups. Compared to the low-risk group, the high-risk group exhibited significantly higher M2-like TAM infiltration (**Figure 5A**). Accordingly, the low-risk group had marked increased expression of immune checkpoint genes than the high-risk group (**Figures 5B, C**).

### High GNA15 expression contributes poor OC prognosis

**Figure 6:**
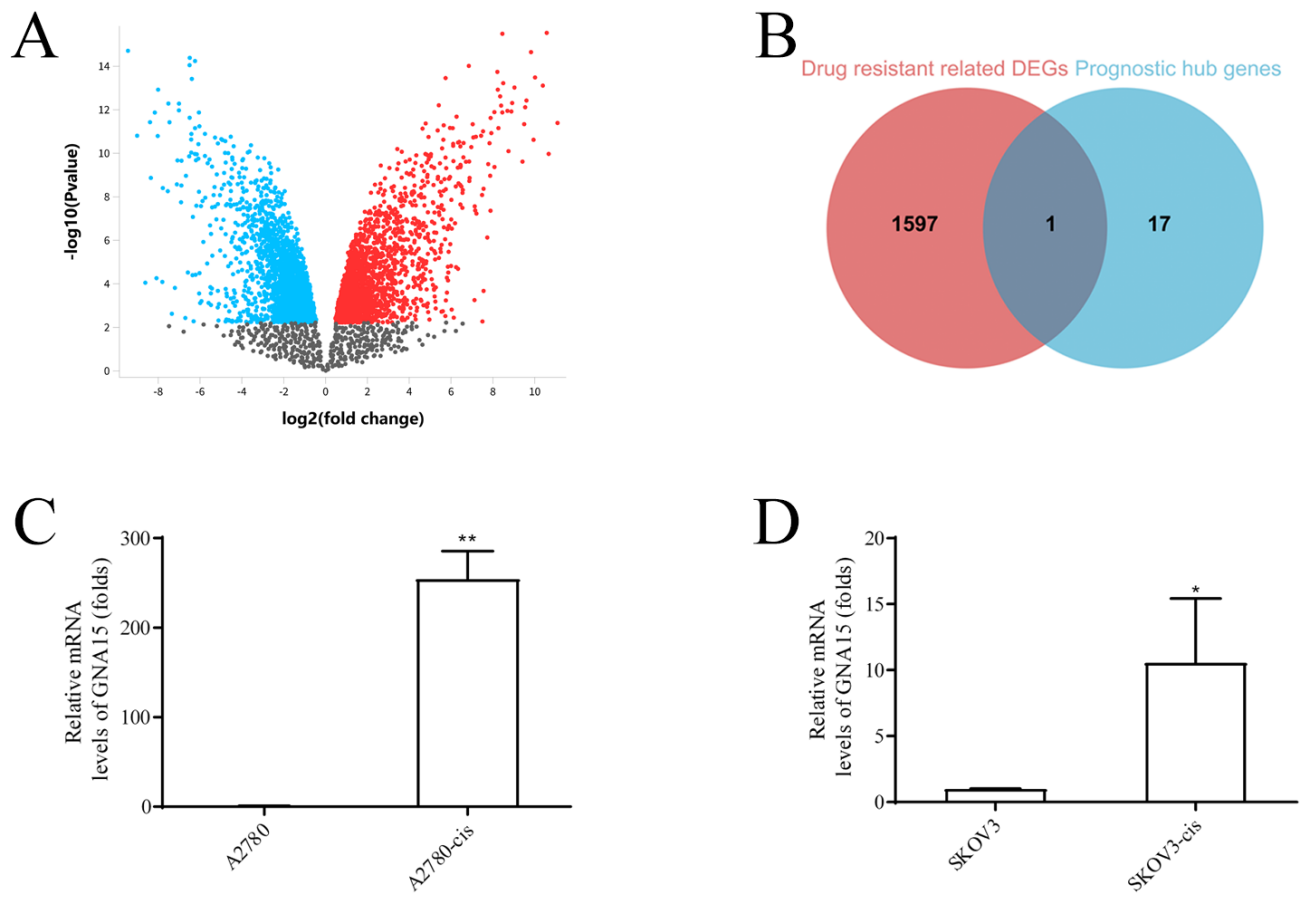
GNA15 is a differentially expressed gene for drug resistance. **(A)** Differential gene expression analysis on the GSE33482 database, red points represent upregulated genes, and green points represent downregulated genes. **(B)** The intersection of 1598 drug resistance related DEGs and 18 prognostic M2-like TAM-related genes. **(C, D)** GNA15 expression between cisplatin-resistant cells of ovarian cancer cell and corresponding OC cells by qRT-PCR analysis. *p < 0.05; **: p < 0.01.

GNA15 was characterized as one of the hub genes most associated with M2-like TAM infiltration in OC, compared to the other genes. In addition, differential gene expression analysis of the GSE33482 database, profiling cisplatin resistance in OC, revealed GNA15 as a differentially expressed gene linked to drug resistance through the intersection of 1598 drug resistance-related DEGs and 18 M2-like TAM-related genes (**Figures 6A, B**). qRT-PCR confirmed GNA15 upregulation in cisplatin-resistant cells (**Figures 6C, D**).

**Figure 7:**
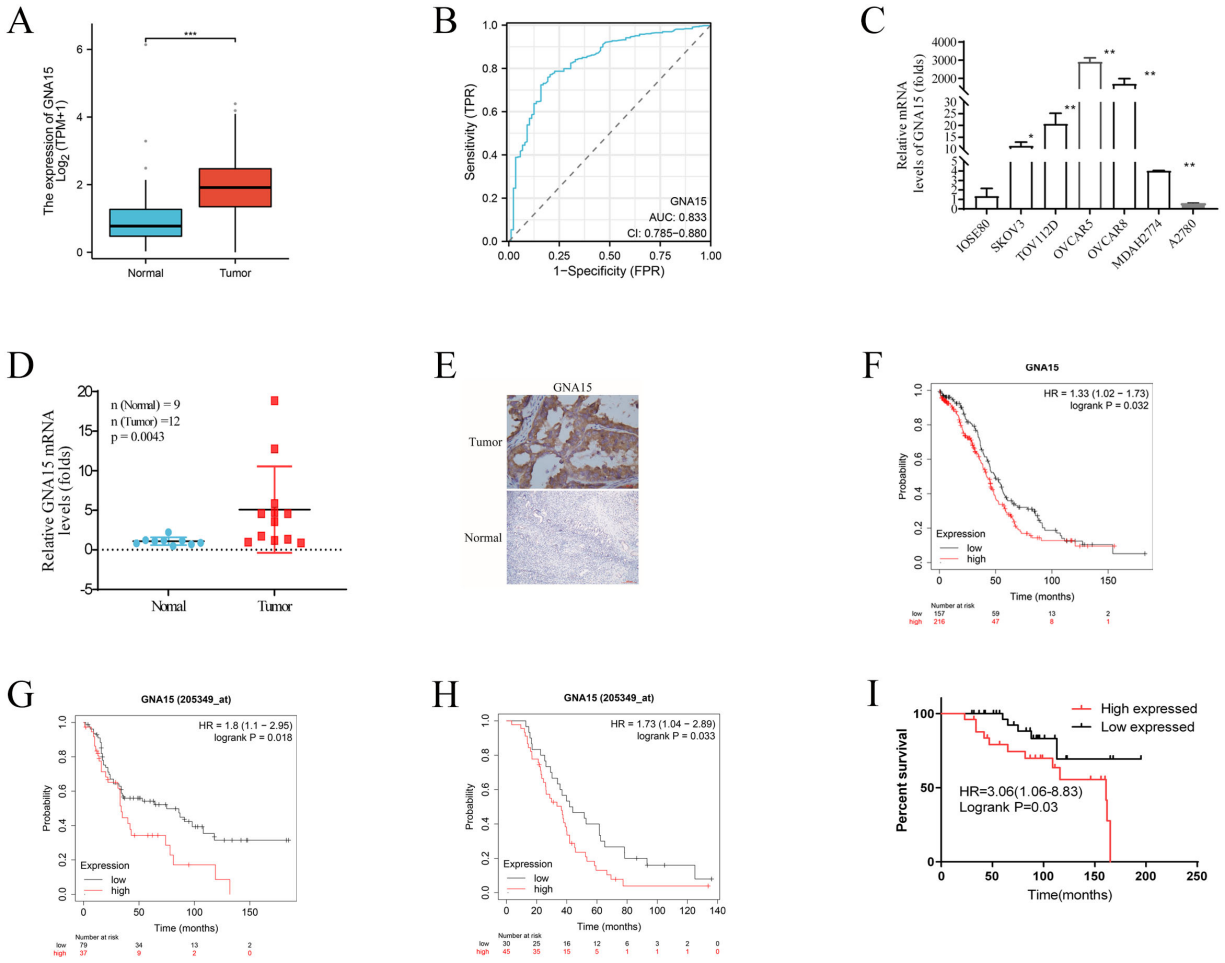
GNA15 expression in normal and cancer tissues. **(A)** RNA-Seq analysis using TCGA-OV samples and matched normal control samples from the GTEx project. **(B)** ROC analysis of GNA15 expression for the discrimination between OC and normal controls using TCGA-OC samples and matched normal control samples from the GTEx project. **(C)** qRT-PCR analysis using OC cells and normal ovarian cells. **(D)** qRT-PCR analysis using clinical OC and normal control samples. **(E)** IHC was performed to detect the expression of GNA15 in OC and normal tissues. **(F–I)** Survival analysis comparing the high and low expression of GNA15 in OC patients based on TCGA-OC database, GSE3149, GSE63885 and our clinical samples. *p < 0.05; **: p < 0.01; *** p < 0.001.

To further investigate the function of GNA15 in OC, we analyzed data from both the TCGA dataset and the GEPIA tool. Our analysis revealed that GNA15 expression was elevated in 10 out of 33 cancer types compared to normal samples (**Supplementary Figure S2A**). Notably, GNA15 expression was significantly higher in OC than in normal tissues (P<0.0001) (**Figure 7A**) and exhibited high sensitivity (**Figure 7B**).

To further verify the results, qRT-PCR and Immunohistochemistry (IHC) assay were used to test GNA15 expression in tumor cell lines and clinical tumor samples including 12 OC and 9 normal control tissues (fresh tissue frozen in liquid nitrogen), and 60 OC samples (the median age of these patients was 52 years and the median follow-up duration for the cohort was 92 months) and 30 normal ovarian samples (paraffin embedded) (**Figures 7C–E**). Consistent with the result from TCGA dataset and GEPIA tool, our results demonstrated that GNA15 was highly expressed in OC than normal samples (*P*<0.05), indicating that GNA15 might have potential functions in carcinogenesis.

The prognostic role of GNA15 was explored based on TCGA-OC database and our clinical samples. The results showed that high GNA15 expression was associated with worse OS [HR=1.33 (1.02-1.74), *P*=0.03] in TCGA-OC database (**Figure 7F**). In addition, we assessed another two OC datasets from GEO database (GSE3149, and GSE63885) to validate the prognostic role of GNA15. Similarly, the results showed high GNA15 expression was associated with worse OS [HR=1.8(1.1-2.95), P=0.018] and [HR=1.73 (1.04-2.89), P=0.03], respectively (**Figures 7G, H**). While in our clinical samples, GNA15 showed a consistent effect in prognosis, elevated GNA15 expression was significantly correlated with a poor prognosis in OC [HR=3.06 (1.06-8.83), *P*=0.03] (**Figure 7I**).

### The relationship between GNA15 and macrophage polarization

In this study, we found a strong correlation between GNA15 and M2 macrophage infiltration abundances using the CIBERSORT algorithm (Spearman r=0.609, *P*<0.001, **Supplementary Figure S3A**). In addition, we investigated the relationship between GNA15 and M2 macrophage biomarkers (MS4A4A, CD163, and VSIG4), demonstrating that elevated GNA15 expression was positively associated with M2 macrophage polarization (**Supplementary Figures S3B–D**). Further, in our clinical samples, we found GNA15 expression was positively related to CD163 expression (**Supplementary Figure S3E**), which suggest GNA15 may related to macrophage polarization.

### Enrichment analysis and tumor heterogeneity analysis of GNA15 expression

Gene set enrichment analysis (GSEA) was performed to explore pathway enrichment between high and low GNA15 expression group. The results showed that immune-associated pathways were more enriched in the high GNA15 expression group, including Chemokine signaling pathway, Leukocyte transendothelial migration, Lysosome, Natural killer, B cell receptor signaling pathway and FcγR mediated phagocytosis (**Supplementary Figure S4A**). The results demonstrated that GNA15 is associated with immune-regulation in tumor microenvironment of OC.

Next, we explored the association between GNA15 expression and tumor heterogeneity. The results demonstrated that GNA15 expression showed a remarkably positive relation with LOH and TMB, while a negative relation with purity (**Supplementary Figures S4B–D**).

### Down-regulating GNA15 *in vitro* decreased OC cell proliferation

**Figure 8:**
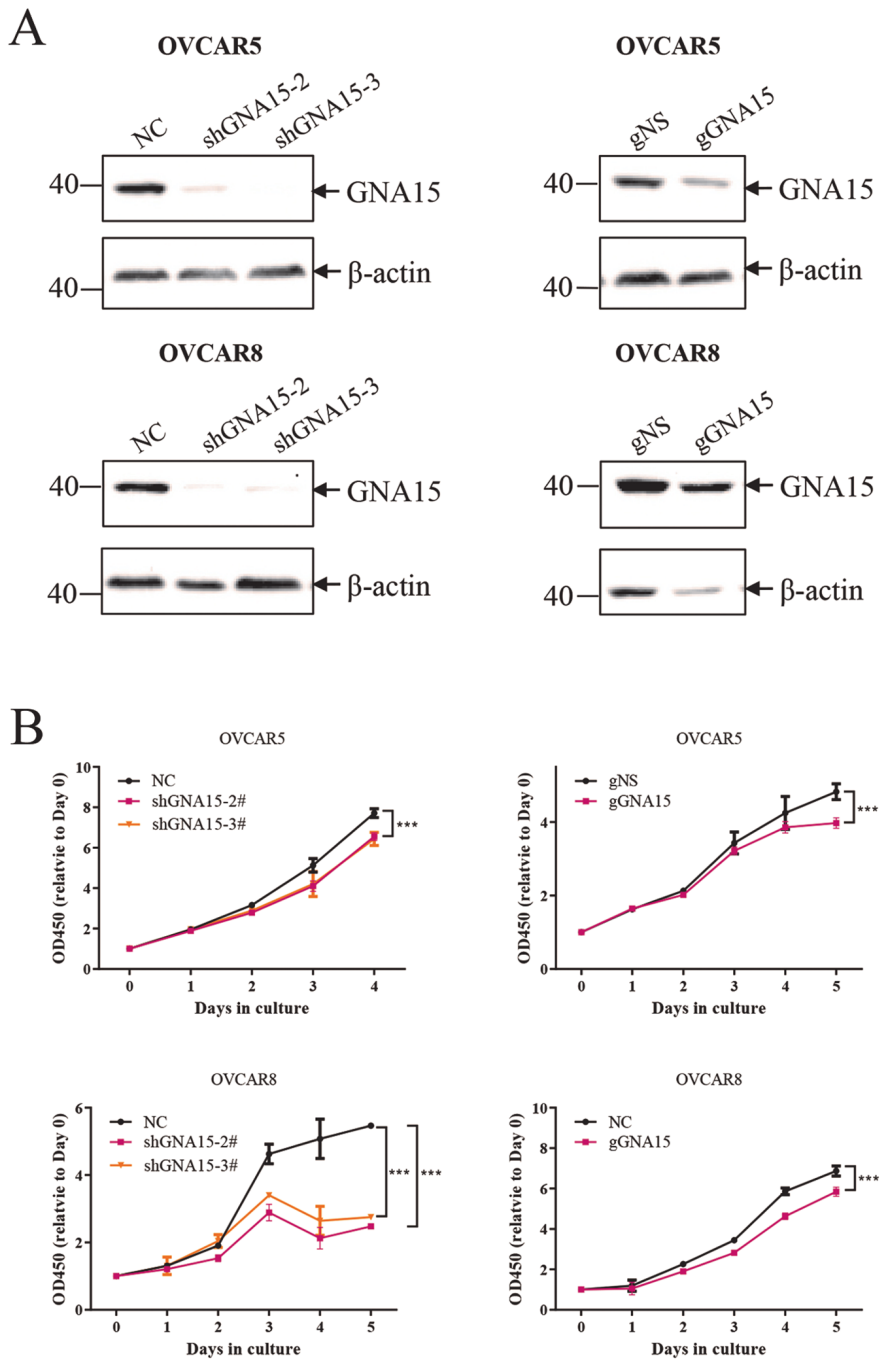
Down-regulating GNA15 *in vitro* decreased ovarian cancer cell proliferation. **(A)** knock down of GNA15 in OVCAR5 and OVCAR8 using ShRNA and CRISRP-Cas9. **(B)** Down-regulating GNA15 significantly decreased ovarian cancer cell proliferation. The protein data were normalized to β-actin. *** p < 0.001.

To determine whether GNA15 plays an important role in cell proliferation, GNA15 was knocked down in OVCAR5 and OVCAR8 using ShRNA and CRISRP-Cas9 (**Figure 8A**). Our data showed that down-regulating GNA15 significantly decreased ovarian cancer cell proliferation (P < 0.0001) (**Figure 8B**).

## Discussion

In the OC microenvironment, TAMs typically adopt an M2-like phenotype, promoting tumor progression by suppressing antitumor immunity and facilitating invasion and metastasis through cytokine and chemokine secretion (28–30). Moreover, TAMs are implicated in OC chemoresistance, as evidenced by their ability to polarize M1-like to M2-like phenotypes upon exposure to cisplatin or carboplatin (31). Therefore, identifying biomarkers associated with M2-like TAMs polarization shows potential for OC treatment strategies. In the present study, we constructed a prognostic model for OC based on an M2-like TAMs gene signature and highlights the initial role of GNA15 in M2-like TAMs polarization. We found: 1) a positive correlation between GNA15 expression and M2-like TAMs infiltration; 2) GNA15 as a predictor of poor prognosis in OC across multiple patient cohorts.

Initially, we employed CIBERSORT (32) to quantify M2-like TAMs infiltration in the TCGA-OC dataset, confirming that high M2-TAMs levels correlate with poorer prognosis in OC. Given M2-like TAMs’ critical roles in prognosis, TME immune modulation, and drug resistance in OC, we conducted a comprehensive analysis. Using WGCNA, we identified genes associated with M2-like TAMs in OC and performed consensus clustering based on 18 prognostic genes. Cluster2 exhibited significantly worse outcomes than Cluster1, with enrichment in pathways such as Fcγ-mediated phagocytosis, Toll-like receptor signaling, B cell receptor signaling, Nod-like receptor signaling, and cancer-related pathways. These pathways are typically associated with unfavorable clinical outcomes. For example, Toll-like receptor activation can promote tumor cell proliferation, inhibit apoptosis (33), and enhance invasion and migration (34), while Fcγ receptors can dampen the efficacy of PD-1 antibodies (35). NOD-like receptor thermal protein domain associated protein 3 (NLRP3)-mediated immune checkpoint regulation also contributes to immune escape in cancers like liver hepatocellular carcinoma and OC, correlating with worse overall survival (36). Immune cell infiltration analysis revealed significantly higher M2 macrophage levels in Cluster2 compared to Cluster1.

Next, we developed an 8-gene signature related to M2-like TAMs. To validate our prognostic model, multivariate regression confirmed the riskscore as an independent prognostic factor in OC. Validation using the GSE140082 dataset further supported the reliability of our signature. Notably, the low-risk group exhibited reduced M2-like TAM infiltration but higher expression of immune checkpoint genes compared to the high-risk group, indicating potential implications for immunotherapy response in OC. Immunotherapy in OC faces challenges due to the immunosuppressive tumor microenvironment, which includes regulatory T cells (Tregs), myeloid-derived suppressor cells (MDSCs), and TAMs. To improve immunotherapy outcomes, identifying TAM-related biomarkers using multi-omics approaches is essential for understanding the immune landscape of OC and developing personalized treatment strategies.

To identify the functions of biomarkers associated with OS and M2-like TAMs infiltration in the OC microenvironment, we investigated 18 prognostic genes intersecting with drug resistance data from GSE33482. We found GNA15 is significantly upregulated in platinum-resistant OC cell lines (A2780-Cis, SKVO3-Cis) and was highly expressed in OC, correlating with poor OS in both TCGA-OC and clinical samples. The following findings highlight the diverse roles of GNA15 across different cellular pathways. It is reported that GNA15 was involved in multiple tumor types by promoting cellular proliferation and inhibiting cellular apoptosis, including liver cancer, pancreatic adenocarcinoma, acute myeloid leukemia, and OC (17–21, 37, 38).

Exploring the role of GNA15 in OC, we confirmed its high expression in OC cells and tissues, consistent with bioinformatics analyses linking GNA15 to poor clinical outcomes. *In vitro* studies demonstrated that knockdown of GNA15 reduced proliferation in OVCAR5 and OVCAR8 cells, suggesting its involvement in OC progression. Existing literature reported that GNA15 is expressed in all myeloid cell lines, suggesting that it may be involved in the regulation of hematopoietic cell differentiation and function (39). Another study reported that GNA demonstrated a high correlation with all identified immune cell subtypes in the ssGSEA algorithm (40). In our study, GSEA analysis revealed enrichment of immune-related pathways, such as the chemokine signaling and FcγR-mediated phagocytosis pathways, in OC samples with high GNA15 expression. Our research has revealed that GNA15 may promote carcinogenesis in OC by modulating TME and enhancing tumor cell proliferation. Specifically, we observed increased infiltration of M2-like TAMs, a key immunosuppressive cell type in the tumor microenvironment, validated by CIBERSORT analysis and confirmed with CD163 immunohistochemical staining (P<0.001). There have also been investigations into the role of GNA15 in regulating cell function. For example, GNA15 downregulation inhibits cell proliferation through the P38 MAPK pathway, a critical regulator of cell survival and growth (21). Moreover, GNA15 has been implicated in the regulation of exosome function, which plays a pivotal role in cell-to-cell communication and shaping the immune microenvironment (22). These findings suggest GNA15 acts as an immunosuppressive factor in OC, potentially influencing M2-like TAM polarization and serving as a prognostic marker.

## Conclusions

Our study has established a prognostic model based on M2-like TAMs to predict survival rates in OC patients, offering valuable insights into the molecular underpinnings of disease progression. Furthermore, we have identified and investigated the potential role of GNA15 within OC cells and the TME, suggesting GNA15 may play a crucial role in immune cell infiltration, macrophage polarization, and tumor progression. This understanding could not only reveal new biomarkers for prognosis but also offer innovative therapeutic targets for immune-based treatments in OC. Moving forward, it is imperative to conduct additional animal experiments and mechanistic studies to elucidate how GNA15 influences macrophage transformation within the OC microenvironment. These endeavors will furnish more robust insights that can be translated into clinical applications.

## Data Availability

Publicly available datasets were analyzed in this study. This data can be found here: https://portal.gdc.cancer.gov/ and https://www.ncbi.nlm.nih.gov/geo/.

## Supplementary material

Supplementary Figure 1 Identification of prognostic M2-like TAMs related genes by WGCNA. **(A)** Samples were clustered and outlier samples were not found. **(B, C)** According to the instructions of the WGCNA package, 5 was selected as the soft threshold power. **(D, E)** Correlation analysis of modules with traits yielded 31 non-gray modules.

Supplementary Figure 2 GNA15 expression in normal and cancer tissues. Comparison of GNA15 mRNA levels across 33 TCGA cancer types and matched normal controls using GEPIA.

Supplementary Figure 3 Correlation of GNA15 expression and M2 macrophages polarization. **(A)** Purity‐corrected Spearman’s correlation between GNA15 expression in OC and M2 macrophages infiltration. **(B-D)** The correlation between GNA15 and molecular biomarkers of M2 macrophages. **(E)** GNA15 expression was positively related to CD163 expression by IHC.

Supplementary Figure 4 The gene enrichment analysis of GNA15 in OC. **(A)** GSEA analysis in GNA15 high and low expressed samples. **(B-D)** Correlation of GNA15 expression and LOH **(B)**, Purity **(C)**, TMB **(D)**.
