# Supplementary figures and images for: GNA15 predicts poor outcomes as a novel biomarker related to M2 macrophage infiltration in ovarian cancer
Source: Front Immunol. 2025 Feb 7;16:1512086. doi: 10.3389/fimmu.2025.1512086 (PMC11842242; doi:10.3389/fimmu.2025.1512086)

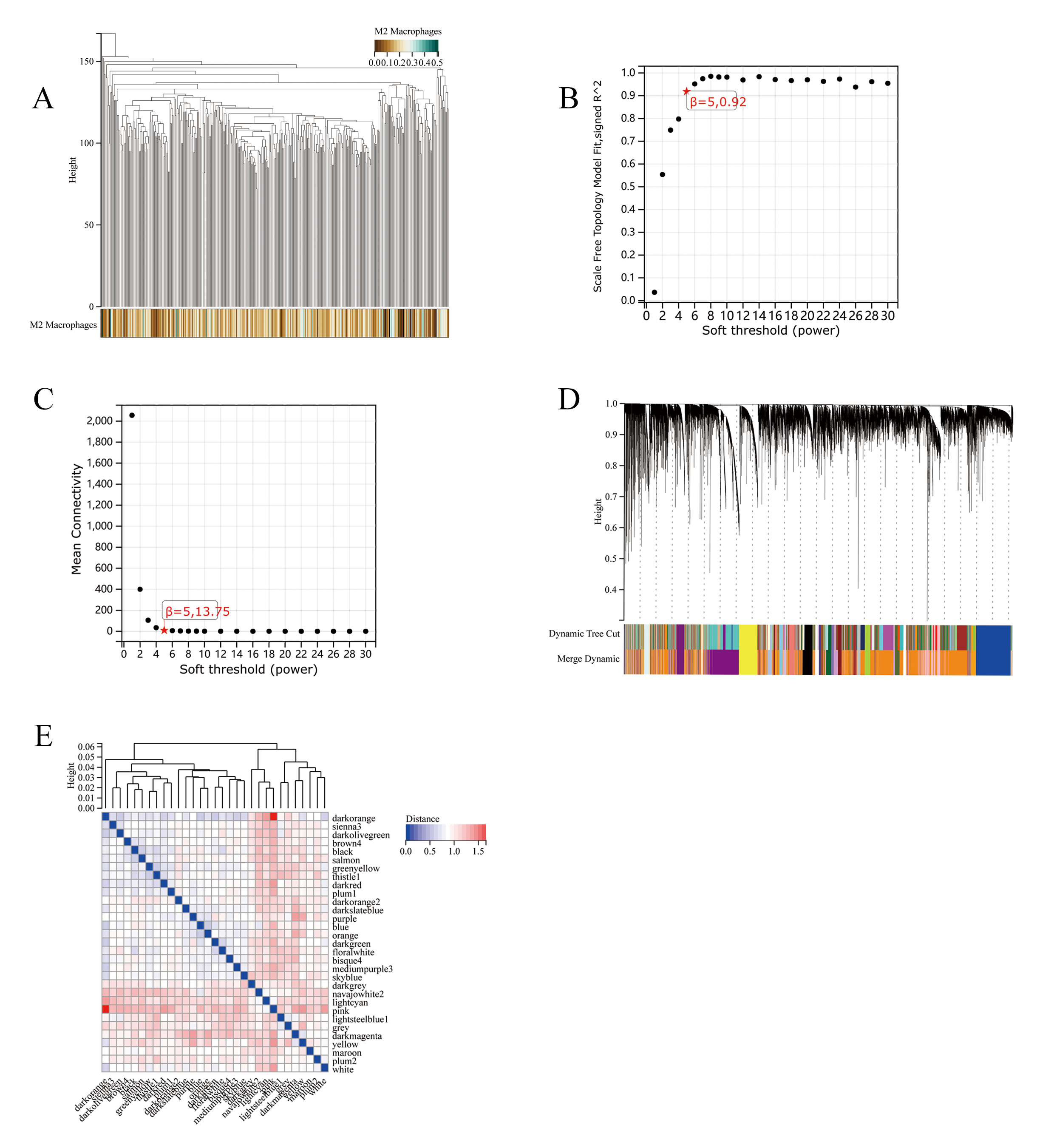

Supplement: Supplementary Figure 1 — Identification of prognostic M2-like TAMs related genes by WGCNA. (A) Samples were clustered and outlier samples were not found. (B, C) According to the instructions of the WGCNA package, 5 was selected as the soft threshold power. (D, E) Correlation analysis of modules with traits yielded 31 non-gray modules. [file Image1.tif]

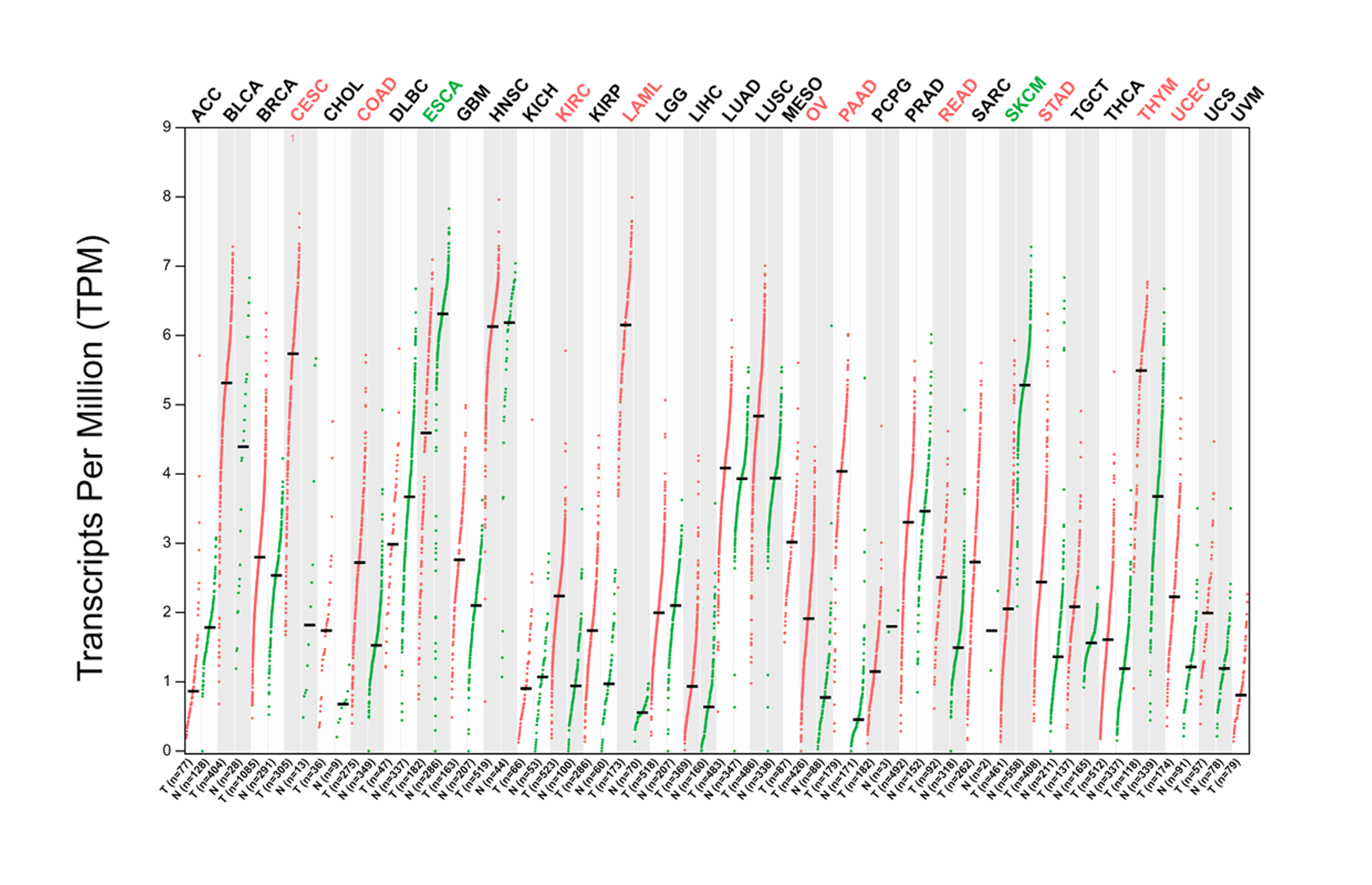

Supplement: Supplementary Figure 2 — GNA15 expression in normal and cancer tissues. Comparison of GNA15 mRNA levels across 33 TCGA cancer types and matched normal controls using GEPIA. [file Image2.tif]

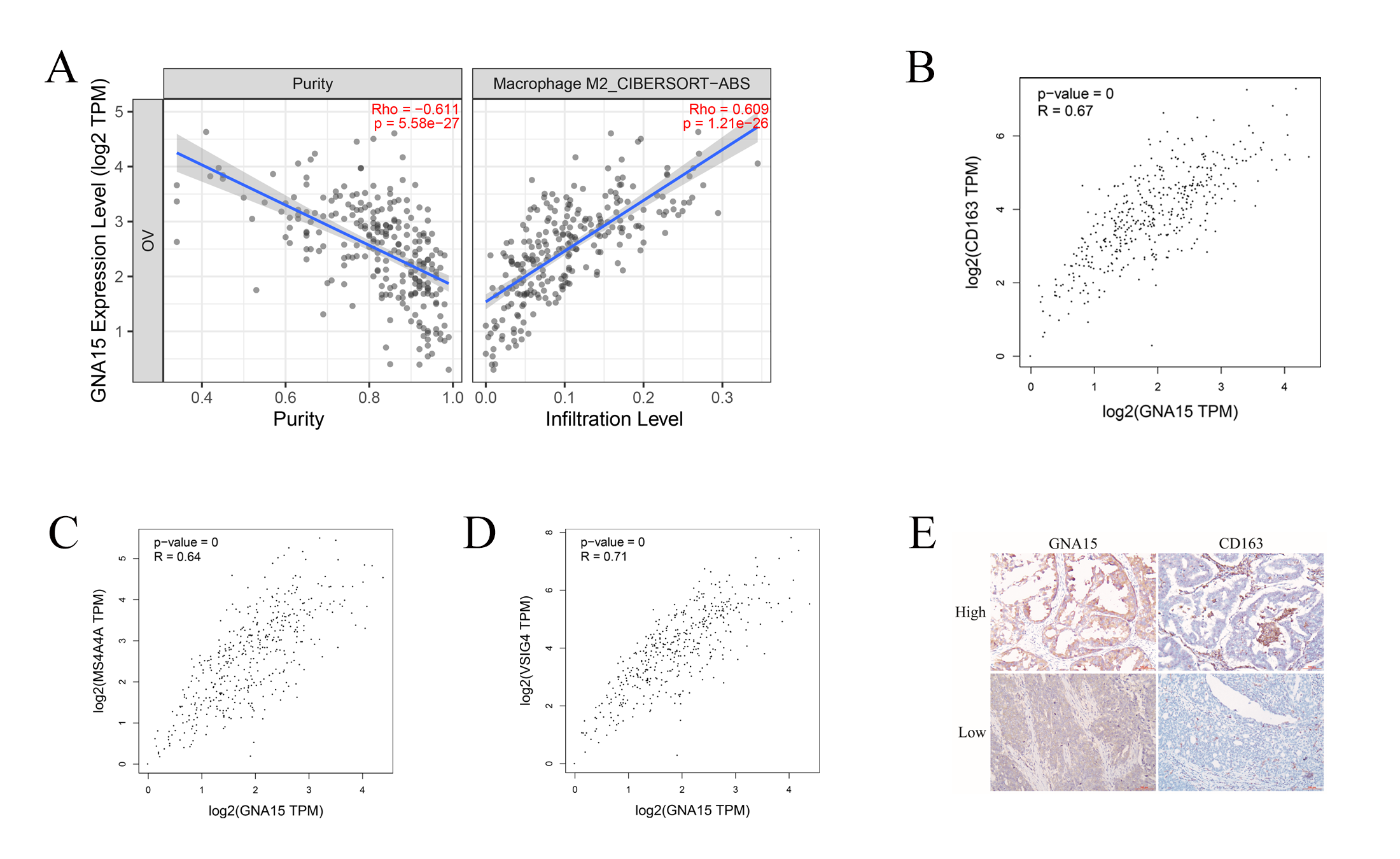

Supplement: Supplementary Figure 3 — Correlation of GNA15 expression and M2 macrophages polarization. (A) Purity‐corrected Spearman’s correlation between GNA15 expression in OC and M2 macrophages infiltration. (B-D) The correlation between GNA15 and molecular biomarkers of M2 macrophages. (E) GNA15 expression was positively related to CD163 expression by IHC. [file Image3.tif]

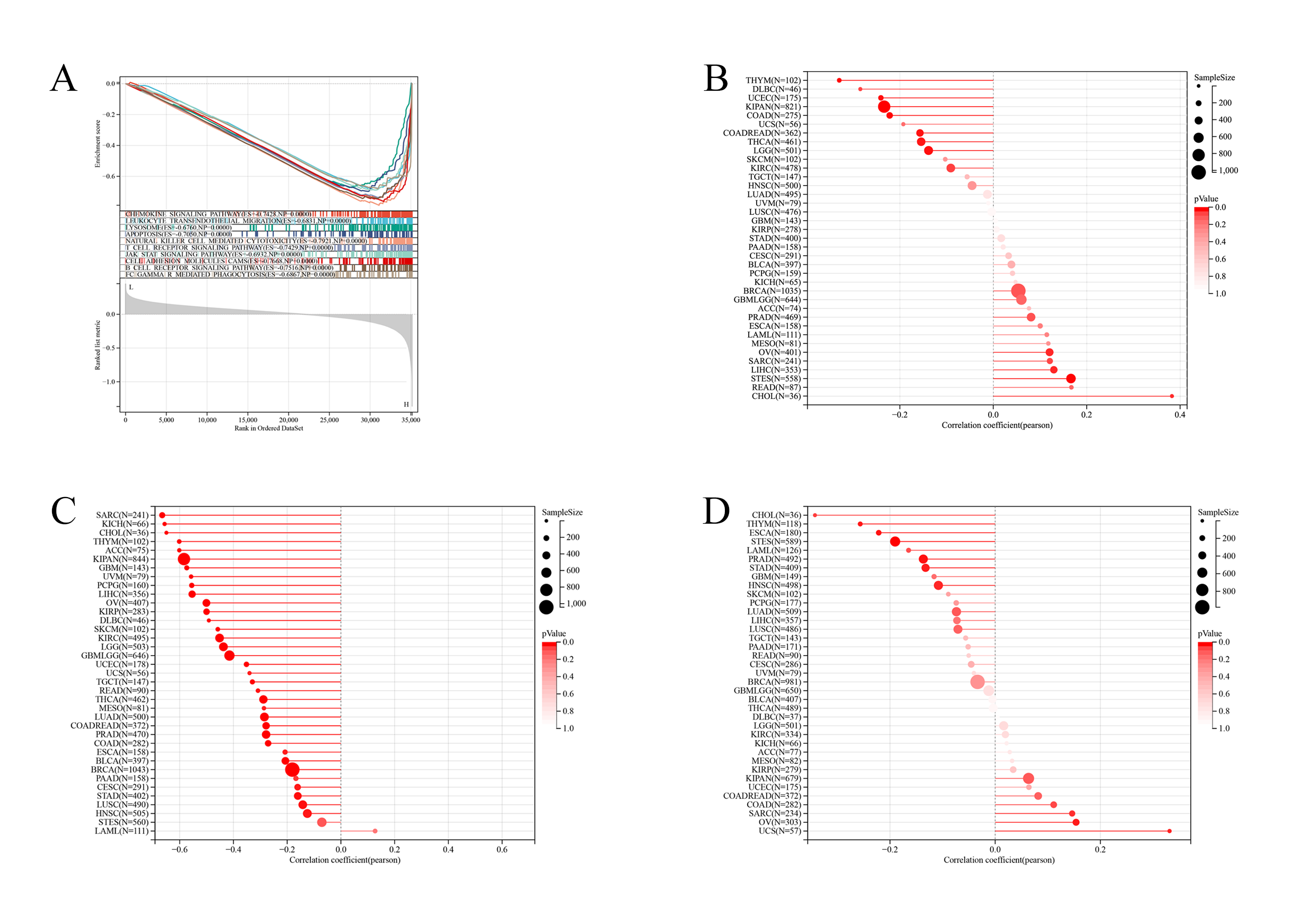

Supplement: Supplementary Figure 4 — The gene enrichment analysis of GNA15 in OC. (A) GSEA analysis in GNA15 high and low expressed samples. (B-D) Correlation of GNA15 expression and LOH (B), Purity (C), TMB (D). [file Image4.tif]
